# Triglyceride-glucose index in the prediction of adverse cardiovascular events in patients with premature coronary artery disease: a retrospective cohort study

**DOI:** 10.1186/s12933-022-01576-8

**Published:** 2022-07-29

**Authors:** Zhenguo Wu, Li Liu, Weiwei Wang, Huiliang Cui, Yerui Zhang, Jiechang Xu, Wencheng Zhang, Tengfei Zheng, Jianmin Yang

**Affiliations:** 1 The Key Laboratory of Cardiovascular Remodeling and Function Research, Chinese Ministry of Education, Chinese National Health Commission and Chinese Academy of Medical Sciences, The State and Shandong Province Joint Key Laboratory of Translational Cardiovascular Medicine, Department of Cardiology, Qilu Hospital of Shandong University, Jinan, China; 2 Department of Cardiology, Boshan District Hospital, Zibo, China

**Keywords:** Triglyceride-glucose index, Premature coronary artery disease, Insulin resistance, Prognosis, Major adverse cardiovascular events, Cohort study

## Abstract

**Background:**

Premature coronary artery disease (PCAD) has become more common in recent years and is often associated with poor outcomes. Triglyceride-glucose (TyG) index is a simple and reliable surrogate for insulin resistance (IR) and is an independent predictor of cardiovascular prognosis. However, the prognostic value of the TyG index in patients with PCAD remains uncertain. Thus, this study aimed to investigate the prognostic value and predictive performance of the TyG index in patients with PCAD.

**Methods:**

A total of 526 young subjects (male < 45 years, female < 55 years) with angiographically proven CAD from January 2013 to December 2018 were included consecutively in this study. Their clinical and laboratory parameters were collected, and the TyG index was calculated as $\mathrm{Ln}[\mathrm{fasting triglyceride }(\mathrm{TG}) (\mathrm{mg}/\mathrm{dL})\times \mathrm{fasting plasma glucose }(\mathrm{FPG}) (\mathrm{mg}/\mathrm{dL})/2]$. The follow-up time after discharge was 40–112 months (median, 68 months; interquartile range, 49‒83 months). The primary endpoint was the occurrence of the major adverse cardiovascular events (MACE), defined as the composite of all-cause death, non-fatal myocardial infarction (MI), coronary artery revascularization, and non-fatal stroke.

**Results:**

The TyG index was significantly associated with traditional cardiovascular risk factors and the Gensini score (GS). Kaplan–Meier survival (MACE-free) curves by tertiles of the TyG index showed statistically significant differences (log-rank test, *p* = 0.001). In the fully adjusted Cox regression model, the Hazard ratio (95% CI) of MACE was 2.17 (1.15–4.06) in tertile 3 and 1.45 (1.11–1.91) for per SD increase in the TyG index. Time-dependent ROC analyses of the TyG for prediction of MACE showed the area under the curves (AUC) reached 0.631 at 3 years, 0.643 at 6 years, and 0.710 at 9 years. Furthermore, adding TyG index to existing risk prediction model could improve outcome prediction [C-statistic increased from 0.715 to 0.719, *p* = 0.007; continuous net reclassification improvement (NRI) = 0.101, *p* = 0.362; integrated discrimination improvement (IDI) = 0.011, *p* = 0.017].

**Conclusion:**

The TyG index is an independent predictor of MACE in patients with PCAD, suggesting that the TyG index has important clinical implications for risk stratification and early intervention of PCAD.

**Supplementary Information:**

The online version contains supplementary material available at 10.1186/s12933-022-01576-8.

## Background

Despite ongoing advances in prevention, diagnosis and treatment, atherosclerosis through its clinical sequelae, coronary artery disease (CAD) and stroke, remains the leading cause of death worldwide [1, 2]. Although CAD can affect any age group, especially the elderly, CAD in young individuals has become more common in recent years [3, 4]. Premature CAD (PCAD) is usually defined as the onset of a cardiovascular event before the age of 45 years in males and 55 years in females [5]. PCAD is an evolving disease and is often associated with poor outcomes. According to Duke Databank for Cardiovascular Disease, half of PCAD patients experienced a substantial evolution of coronary atherosclerosis within 10 years, and 1 in 5 patients died prematurely [6].

There is growing evidence that insulin resistance (IR), which is a prominent characteristic of the metabolic syndrome and type 2 diabetes mellitus (DM), may also be involved in the pathogenesis of CAD [7–9]. Metabolic syndrome has been proven a risk factor for cardiovascular morbidity and mortality beyond traditional risk factors in young adults [10–12]. Moreover, a previous study showed that baseline DM was the only variable independently associated with a first ischemic recurrence among traditional risk factors in PCAD patients [6]. All evidence indicated that IR may play a crucial role in the progression of PCAD and as a risk factor for poor outcomes.

Given the inherent limitations of traditional IR assessment methods (such as the hyperinsulinemic-euglycemic clamp technique and homeostasis model assessment for IR) [13], the triglyceride-glucose (TyG) index has been evaluated as a reliable surrogate for IR [14, 15]. Cohort studies performed in America, Europe and China have found the TyG index as an independent risk factor for the incidence of cardiovascular disease (CVD) [16–19]. Tehran Lipid and Glucose Study (TLGS) reported that TyG was independently associated with incident CVD, especially among the younger age group (< 60 years) [20]. Recent studies also showed that the TyG index could predict the in-stent restenosis and prognosis of elderly acute coronary syndrome (ACS) patients [21, 22]. However, little research has investigated the TyG index in patients with PCAD.

Therefore, we aimed to explore whether the TyG index has a prognostic value for major adverse cardiovascular events (MACE) in PCAD patients, and further explore associations between the TyG index and different cardiovascular events in different subgroups.

## Methods

### Study design and patients

This study complied with the Declaration of Helsinki and was approved by the Ethics Review Committee of Qilu Hospital of Shandong University (Approval No. 2018–055). As this was a retrospective cohort study and the follow-up was performed by phone, the ethics committee permitted verbal consent.

**Fig. 1:**
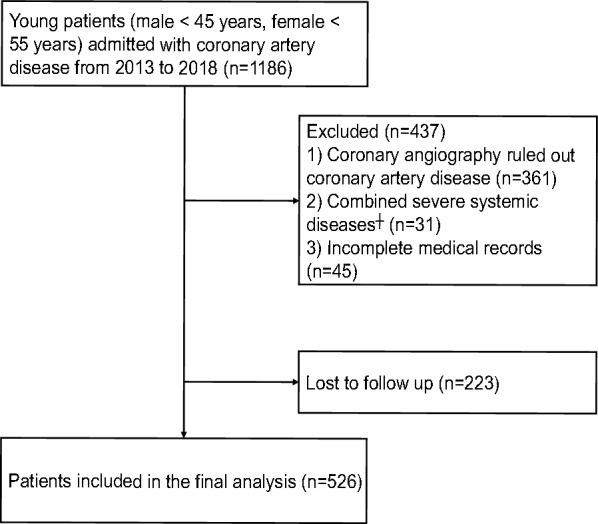
Flow diagram of patient selection. ^┼^ Including severe valvular heart disease, decompensated heart failure, non-ischemic dilated cardiomyopathy, severe renal or hepatic disease, acute infection and/or inflammation, malignancy, hematologic disease or autoimmune disease

This study was a single-center, retrospective cohort study. From January 2013 to December 2018, 1186 consecutive young patients (male < 45 years, female < 55 years) with the first manifestation of CAD underwent coronary angiography at Qilu Hospital of Shandong University, and 825 of them with angiographically proven CAD. CAD was defined as the presence of obstructive stenosis of > 50% of the vessel lumen diameter in any of the main coronary arteries, including the left main coronary artery (LM), left anterior descending artery (LAD), left circumflex coronary artery (LCX) and right coronary artery (RCA), or main branches of the vascular system. Those with severe valvular heart disease, decompensated heart failure, non-ischemic dilated cardiomyopathy, severe renal or hepatic disease (serum creatinine > 1.4 mg/dL or liver function parameters > 3 $\times$ upper normal value), acute infection and/or inflammation, malignancy, hematologic disease, autoimmune disease or those having incomplete medical records were excluded. A total of 749 patients were enrolled in this study. Patients were followed up from March 2022 to April 2022 by telephone and 526 (70.2%) provided verbal consent and completed the telephone follow-up (Fig. 1).

### Data collection and definitions

Clinical data were collected from medical records by trained clinicians who were blinded to the study aim. The data included patients’ general conditions [age, gender, body mass index (BMI), left ventricular ejection fraction (LVEF), admission events, number of lesions and Gensini score (GS)], cardiovascular risk factors [current smoking, family history of CAD (FH-CAD), DM and hypertension], laboratory tests [fasting plasma glucose (FPG), total cholesterol (TC), triglyceride (TG), low‐density lipoprotein cholesterol (LDL-C), high-density lipoprotein cholesterol (HDL-C), serum creatinine (SCr) and uric acid (UA)] and basic cardiovascular medication information [antiplatelet drugs, statins, beta-blockers, angiotensin-converting enzyme inhibitors (ACEI)/angiotensin receptor blockers (ARB)]. Peripheral venous blood samples were collected early in the morning after overnight fasting (8 h minimum) and the levels of blood biochemical indicators were measured. BMI was defined as weight (kg) divided by the square of height (m^2^). Myocardial infarction (MI) was defined according to clinical symptoms, electrocardiogram, and cardiac biomarkers [23]. According to coronary angiography results, the severity of CAD was evaluated by the GS [24], and multivessel disease was defined as $\ge$ 50% diameter stenosis in at least 2 major coronary arteries. FH-CAD was defined as a history of CAD in a first-degree relative < 55 years (male) or < 65 years (female). DM was defined as FPG $\ge$ 7.0 mmol/L, random blood glucose (RBG) $\ge$ 11.1 mmol/L, 2 h plasma glucose after oral glucose tolerance test (OGTT)$\ge$ 11.1 mmol/L, or use of insulin or oral hypoglycemic agents. Hypertension was defined as systolic blood pressure $\ge$ 140 mmHg and/or diastolic blood pressure $\ge$ 90 mmHg, or on antihypertensive medication. Hyperlipidemia was defined as ICD-10 code E78 with lipid-lowering drugs or TC ≥ 240 mg/dL [25]. The estimated glomerular filtration rate (eGFR) was calculated using SCr by the Chinese modified Modification of Diet in Renal Disease equation as following [26]:$\mathrm{eGFR }\,(\mathrm{mL}/\mathrm{min}/1.73\mathrm{m}^{2}) =175\times {\mathrm{SCr}\,(\mathrm{mg}/\mathrm{dL})}^{-1.234}\times {\mathrm{age }\left(\mathrm{year}\right)}^{-0.179}\times 0.79\, (\mathrm{if\, female})$. The TyG index was calculated as $\mathrm{Ln}[\mathrm{fasting TG }(\mathrm{mg}/\mathrm{dL})\times \mathrm{FPG }(\mathrm{mg}/\mathrm{dL})/2]$ [27].

### Endpoints

The primary endpoint of our study was the occurrence of MACE, defined as the composite of all-cause death (cardiovascular or non-cardiovascular death), non-fatal MI, coronary artery revascularization (PCI or CABG), and non-fatal stroke (ischemic, hemorrhagic, or unspecified). The secondary endpoints included all-cause death, non-fatal MI, coronary artery revascularization, and non-fatal stroke.

### Statistical analysis

Statistical analysis was performed using SPSS version 25.0 (SPSS, Chicago, IL) and R software version 4.1.3 (R Foundation for Statistical Computing). Subjects were classified according to the occurrence of MACE during the follow-up and the tertile of the TyG index. Continuous variables were presented as mean ± standard deviation (SD) or median with the 25th and 75th percentiles, as appropriate, and compared using the Student’s t-test or ANOVA test in case of Gaussian distribution, or Mann–Whitney U test or Kruskal–Wallis H test in case of non-Gaussian distribution. Categorical variables were expressed with counts and percentages and compared using the chi-square test or Fisher exact test. The associations between the TyG index and traditional cardiovascular risk factors were assessed using Pearson or Spearman correlation analysis. Kaplan–Meier event-free survival curves were generated and the significance was assessed by log-rank tests. Variables were analyzed by univariate Cox regression analysis. To further determine whether the TyG index was an independent predictor for the occurrence of MACE, multivariate Cox proportional hazards regression was performed. We built 3 regression models of increasing confounders: model 1 was adjusted for age and gender, model 2 was the partially adjusted model that was adjusted for variables with *p* < 0.05 in univariate analysis; and model 3 was the fully adjusted model that was adjusted for age, gender, BMI, LVEF, admission for MI, multivessel disease, GS, current smoking, current drinking, FH-CAD, DM, hypertension, hyperlipidemia, TC, LDL-C, HDL-C, eGFR, UA, antiplatelet drugs, statins, beta-blockers, ACEI/ARB and hypoglycemic drugs. The TyG index was entered into the models as continuous variables and categorical variables (the tertile of the TyG index) respectively. The TyG index was further standardized to determine the predictive value of the TyG index per SD increase. The variance inflation factor (VIF) of the variables included in the models was calculated to avoid the result deviation caused by multicollinearity. We did not find evidence of collinearity in the models, given the VIF of < 10. We also performed subgroup analysis based on gender, admission events, DM, hypertension and hyperlipidemia to determine whether the association between TyG index and MACE differed across various subgroups and *p* for interaction was calculated. Time-dependent receiver operating characteristic (ROC) curves were performed and the area under the curve (AUC) was used to estimate the predictive value of the TyG index. To evaluate whether an increased TyG index had incremental predictive value for MACE, we compared the fully adjusted model (model 3) with and without the TyG index, and C-statistics, continuous net reclassification improvement (NRI) and integrated discrimination improvement (IDI) were obtained. A *p*-value of less than 0.05 was considered to be statistically significant.

## Results

### Baseline characteristics

A total of 526 patients with PCAD were enrolled in this study, with an average age of 44.48 ± 6.30 years, and 316 (60.2%) patients were male. Baseline characteristics of participants with and without MACE were shown in Table 1. Patients in whom a cardiovascular event developed tended to be smokers (*p* = 0.035), drinkers (*p* = 0.024) or to have multivessel disease (*p* < 0.001), diabetes (*p* < 0.001) or hypertension (*p* = 0.018). Significant differences could also be found for GS (*p* = 0.035), FPG (*p* < 0.001), TG (*p* = 0.027), LDL-C (*p* = 0.029) and use of hypoglycemic drugs (*p* < 0.001). Moreover, the patients with MACE presented a significantly higher level of the TyG index than those without event (8.94 ± 0.52 vs. 8.72 ± 0.57, *p* < 0.001) (Table 1).

**Table 1:** Baseline characteristics of the study population according to the occurrence of MACE

Variables	Total (n = 526)	Without event (n = 425)	With event (n = 101)	*p*-value
General conditions				
Age (years)	44.48 ± 6.30	44.60 ± 6.20	44.01 ± 6.75	0.400
Male, n (%)	316(60.1)	258 (60.7)	58 (57.4)	0.545
BMI (kg/m^2^)	26.90 ± 3.36	26.78 ± 3.17	27.42 ± 4.04	0.138
LVEF (%)	59.54 ± 9.99	59.56 ± 10.11	59.42 ± 9.53	0.893
Admission for MI, n (%)	153 (29.1)	121 (28.5)	32 (31.7)	0.523
GS	36.75 (22.00–61.00)	35.00 (21.50–60.00)	47.00 (25.00–64.00)	**0.035**
Multivessel disease, n (%)	233 (44.3)	170 (40.0)	63 (62.4)	** < 0.001**
Risk factors, n (%)				
Current smoking	148 (28.1)	111 (26.1)	37 (36.6)	**0.035**
Current drinking	99 (18.8)	72 (16.9)	27 (26.7)	**0.024**
FH-CAD	144 (27.4)	110 (25.9)	34 (33.7)	0.115
DM	103 (19.6)	70 (16.5)	33 (32.7)	** < 0.001**
Hypertension	299 (56.8)	231 (54.4)	68 (67.3)	**0.018**
Hyperlipidemia	176 (33.5)	140 (32.9)	36 (35.6)	0.605
Laboratory test				
FPG (mmol/L)	4.94 (4.51–5.88)	4.91 (4.47–5.65)	5.40 (4.64–7.29)	** < 0.001**
TC (mmol/L)	4.01 (3.36–4.83)	3.97 (3.34–4.79)	4.15 (3.49–4.94)	0.293
TG (mmol/L)	1.43 (1.10–1.99)	1.41 (1.08–1.97)	1.58 (1.26–2.05)	**0.027**
LDL-C (mmol/L)	2.49 (1.94–3.13)	2.45 (1.92–3.08)	2.65 (2.07–3.23)	**0.029**
HDL-C (mmol/L)	1.18 ± 0.25	1.18 ± 0.26	1.15 ± 0.22	0.299
eGFR (mL/min/1.73m^2^)	118.06 ± 25.99	117.76 ± 26.11	119.34 ± 25.54	0.584
UA (μmol/L)	317.00(257.00–372.25)	320.00(258.50–371.00)	304.00(252.00–388.00)	0.637
Cardiovascular medications, n (%)				
Antiplatelet drugs	497 (94.5)	401 (94.4)	96 (95.0)	0.783
Stains	501 (95.2)	408 (96.0)	93 (92.1)	0.160
Beta-blockers	322(61.2)	265 (62.4)	57 (56.4)	0.273
ACEI/ARB	246 (46.8)	196 (46.1)	50 (49.5)	0.540
Hypoglycemic drugs	90 (17.1)	60 (14.1)	30 (29.7)	** < 0.001**
TyG index	8.76 ± 0.56	8.72 ± 0.57	8.94 ± 0.52	** < 0.001**

Data were given as mean ± SD, median with interquartile range or n (%)

*MACE* major adverse cardiovascular events, *BMI* body mass index, *LVEF* left ventricle ejection fraction, *MI* myocardial infarction, *GS* Gensini score, *FH-CAD* family history of coronary artery disease, *DM* diabetes mellitus, *FPG* fasting plasma glucose, *TC* total cholesterol, *TG* triglyceride, *LDL-C* low-density lipoprotein-cholesterol, *HDL-C* high-density lipoprotein-cholesterol, *eGFR* estimated glomerular filtration rate, *UA* uric acid, *ACEI* angiotensin-converting enzyme inhibitors, *ARB* angiotensin receptor blockers, *TyG* index, triglyceride-glucose index

*p* values in bold are < 0.05

As shown in Table 2, the patients were divided into 3 groups according to the tertile of the TyG index (tertile 1: n = 175, TyG index < 8.51; tertile 2: n = 176, 8.51 $\le$ TyG index < 8.96; and tertile 3: n = 175, TyG index $\ge$ 8.96). There were significant differences among the three groups in terms of age, BMI, GS, FPG, TC, TG, LDL-C, and the proportion of admission for MI, FH-CAD, DM, hyperlipidemia, ACEI/ARB use, hypoglycemic drugs use, MACE, and coronary artery revascularization. No significant difference was found in the other indicators and secondary endpoints (Table 2).

**Table 2:** Baseline characteristics of the study population according to the tertiles of the TyG index

Variables	Tertile 1 (n = 175)	Tertile 2 (n = 176)	Tertile 3 (n = 175)	*p*-value
TyG index	8.18 ± 0.26	8.72 ± 0.13	9.39 ± 0.36	** < 0.001**
General conditions				
Age (years)	45.22 ± 6.27	43.35 ± 6.03	44.89 ± 6.48	**0.012**
Male, n (%)	98 (56.0)	117 (66.5)	101 (57.7)	0.099
BMI (kg/m^2^)	26.25 ± 2.98	26.98 ± 3.49	27.47 ± 3.49	**0.003**
LVEF (%)	59.13 ± 10.84	59.81 ± 9.80	59.66 ± 9.32	0.799
Admission for MI, n (%)	36 (20.6)	63 (35.8)	54 (30.9)	**0.006**
GS	31.00 (17.00–52.00)	36.50 (22.00–59.50)	45.00 (27.00–65.00)	** < 0.001**
Multivessel disease, n (%)	73 (41.7)	82 (46.6)	78 (44.6)	0.653
Risk factors, n (%)				
Current smoking	43 (24.6)	51 (29.0)	54 (30.9)	0.406
Current drinking	31 (17.7)	39 (22.2)	29 (16.6)	0.367
FH-CAD	27 (15.4)	48 (27.3)	69 (39.4)	** < 0.001**
DM	11 (6.3)	24 (13.6)	68 (38.9)	** < 0.001**
Hypertension	94 (53.7)	107 (60.8)	98 (56.0)	0.393
Hyperlipidemia	15 (8.6)	63 (35.8)	98 (56.0)	** < 0.001**
Laboratory test				
FPG (mmol/L)	4.54 (4.17–4.89)	4.91 (4.57–5.73)	5.81 (5.06–7.97)	** < 0.001**
TC (mmol/L)	3.73 (3.12–4.26)	4.02 (3.37–4.77)	4.57 (3.70–5.35)	** < 0.001**
TG (mmol/L)	1.01 (0.84–1.19)	1.49 (1.32–1.80)	2.21 (1.79–2.71)	** < 0.001**
LDL-C (mmol/L)	2.27 (1.71–2.69)	2.54 (2.00–3.13)	2.85 (2.07–3.54)	** < 0.001**
HDL-C (mmol/L)	1.20 ± 0.26	1.16 ± 0.23	1.17 ± 0.27	0.495
eGFR (mL/min/1.73m^2^)	118.55 ± 24.11	116.47 ± 27.53	119.18 ± 26.25	0.595
UA (μmol/L)	310.26 ± 80.11	324.40 ± 87.37	331.10 ± 95.52	0.078
Cardiovascular medications, n (%)				
Antiplatelet drugs	164 (93.7)	168 (95.5)	165 (94.3)	0.767
Stains	168 (96.0)	164 (93.2)	169 (96.6)	0.279
Beta-blockers	103 (58.9)	111 (63.1)	108 (61.7)	0.711
ACEI/ARB	62 (35.4)	91 (51.7)	93 (53.1)	**0.001**
Hypoglycemic drugs	11 (6.3)	19 (10.8)	60 (34.3)	** < 0.001**
Outcomes, n (%)				
MACE	20 (11.4)	36 (20.5)	45 (25.7)	**0.003**
All-cause death	2 (1.1)	1 (0.6)	3 (1.7)	0.543
Cardiovascular death	1 (0.6)	1 (0.6)	3 (1.7)	0.544
Non-fatal MI	5 (2.9)	10 (5.7)	14 (8.0)	0.108
Coronary artery revascularization	12 (6.9)	23 (13.1)	27 (15.4)	**0.037**
Non-fatal stroke	1 (0.6)	2 (1.1)	1 (0.6)	1.000

Data were given as mean ± SD, median with interquartile range or n (%)

*TyG* index, triglyceride-glucose index, *BMI* body mass index, *LVEF* left ventricle ejection fraction, *MI* myocardial infarction, *GS* Gensini score, *FH-CAD* family history of coronary artery disease, *DM* diabetes mellitus, *FPG* fasting plasma glucose, *TC* total cholesterol, *TG* triglyceride, *LDL-C* low-density lipoprotein-cholesterol, *HDL-C* high-density lipoprotein-cholesterol, *eGFR* estimated glomerular filtration rate, *UA* uric acid, *ACEI* angiotensin-converting enzyme inhibitors, *ARB* angiotensin receptor blockers, *MACE* major adverse cardiovascular events

*p* values in bold are < 0.05

### Correlations between the TyG index and cardiovascular risk factors

The associations between the TyG index and cardiovascular risk factors were examined using Spearman or Pearson correlation analysis. As shown in Table 3, the TyG index was positively associated with BMI, GS, FPG, TC, TG LDL-C and UA (*p* < 0.05). No significant correlation was observed between TyG and age, LVEF, HDL-C and eGFR (Table 3).

**Table 3:** Correlations between the TyG index and cardiovascular risk factors

Variables	Correlation coefficient (r)	*p*-value
Age (years)	− 0.010^┼^	0.821
BMI (kg/m^2^)	0.127^┼^	**0.003**
LVEF (%)	− 0.027^┼^	0.532
GS	0.181^§^	** < 0.001**
FPG (mmol/L)	0.582^§^	** < 0.001**
TC (mmol/L)	0.316^§^	** < 0.001**
TG (mmol/L)	0.867^§^	** < 0.001**
LDL-C (mmol/L)	0.286^§^	** < 0.001**
HDL-C (mmol/L)	− 0.022^┼^	0.615
eGFR (mL/min/1.73m^2^)	− 0.033^┼^	0.455
UA (μmol/L)	0.141^§^	**0.001**

*TyG index* triglyceride-glucose index, *BMI* body mass index, *LVEF* left ventricle ejection fraction, *GS* Gensini score, *FPG* fasting plasma glucose, *TC* total cholesterol, *TG* triglyceride, *LDL-C* low-density lipoprotein-cholesterol, *HDL-C* high-density lipoprotein-cholesterol, *eGFR* estimated glomerular filtration rate, *UA* uric acid

*p* values in bold are < 0.05

^┼^Pearson correlation analysis

^§^Spearman correlation analysis

### TyG index and cardiovascular events

**Fig. 2:**
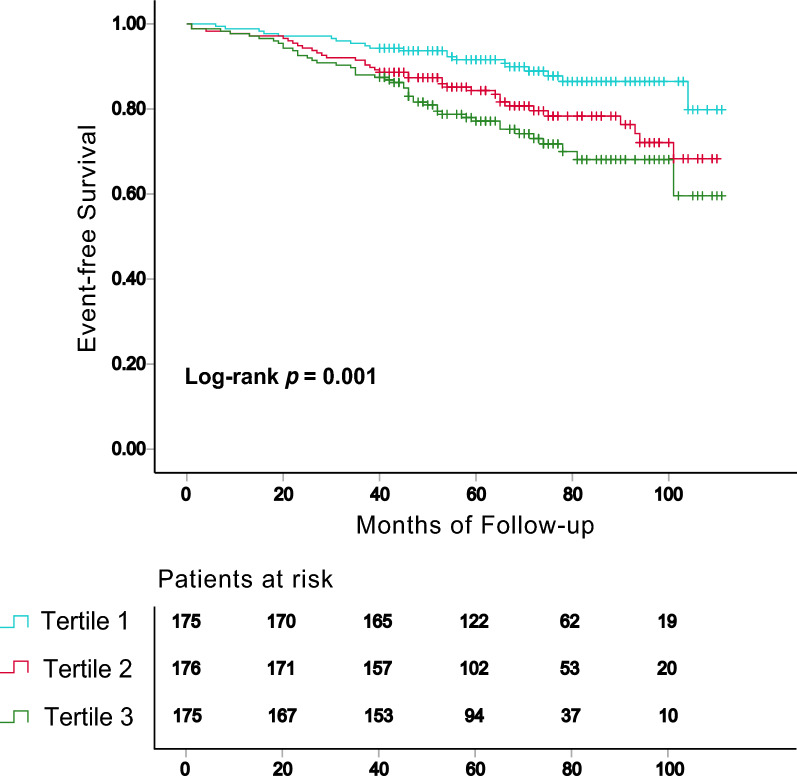
Kaplan–Meier survival curve for MACE across the TyG index tertiles

The follow-up time of this study was 40–112 months (median, 68 months; interquartile range, 49‒83 months). During the follow-up, 101 (19.2%) MACEs were recorded, including 6 (1.1%) all-cause death, 29 (5.5%) non-fatal MI, 62 (11.8%) coronary artery revascularization and 4 (0.8%) non-fatal stroke. To show the outcomes of patients with different levels of the TyG index, we generated Kaplan–Meier survival plots (Fig. 2). As shown in Fig. 2, the cumulative incidence of MACE increased incrementally across tertiles of the TyG index (log-rank test, *p* = 0.001). Univariate Cox regression analysis was used to identify the factors associated with MACE. As presented in Table 4, BMI, multivessel disease, current smoking, DM, hypertension, FPG, LDL-C, statins use, hypoglycemic drugs use and the TyG index were found to be risk factors for MACE. The unadjusted HR (95% CI) for risk of MACE with per SD increase in the TyG index was 1.46 (1.22–1.76) (Table 4). Multivariate Cox proportional hazards regression analysis showed that the TyG index, whether considered as a categorical or continuous variable, remained significant after adjusting for confounders. For per SD increase in the TyG index, the risk of incident MACE increased by 29% (HR = 1.29; 95% CI 1.04–1.60) in the partially adjusted regression model. Compared with subjects in the lowest tertile, the partially adjusted HR for MACE was 1.58 (95% CI 0.91–2.75) and 1.99 (95% CI 1.12–3.52) in the middle and highest tertile, respectively. The increased risk of MACE from tertile 1 to tertile 3 was statistically significant (*p* for trend = 0.020). A similar pattern was observed in fully adjusted model (Per SD increase: HR = 1.45, 95% CI 1.11–1.91; Tertile 2: HR = 1.55, 95% CI 0.87–2.78; Tertile 3: HR = 2.17, 95% CI 1.15–4.06; *p* for trend = 0.016) (Table 5). Moreover, the sensitivity analysis that excluded patients with a history of lipid-lowering or hypoglycemic usage and non-cardiovascular death showed results consistent with the primary analysis (Additional file 1: Table S1).

**Table 4:** Univariate Cox regression analyses for MACE

Variables	HR	95%CI	*p*-value
Age	0.99	0.96–1.02	0.410
Male	0.84	0.57–1.25	0.393
BMI	1.07	1.01–1.13	**0.033**
LVEF	0.84	0.12–5.97	0.861
Admission for MI	1.10	0.72–1.67	0.655
Multivessel disease	1.71	1.14–2.57	**0.009**
GS	1.01	1.00–1.01	0.119
Current Smoking	1.56	1.04–2.32	**0.032**
Current Drinking	1.25	0.80–1.95	0.335
FH-CAD	1.46	0.97–2.21	0.073
DM	2.16	1.43–3.28	** < 0.001**
Hypertension	1.75	1.16–2.66	**0.008**
Hyperlipidemia	1.20	0.80–1.80	0.390
FPG	1.16	1.09–1.25	** < 0.001**
TC	1.13	0.98–1.30	0.091
TG	1.11	0.92–1.34	0.286
LDL-C	1.23	1.03–1.48	**0.024**
HDL-C	0.64	0.29–1.42	0.272
eGFR	1.00	0.99–1.01	0.672
UA	1.00	1.00–1.00	0.759
Antiplatelet drugs	0.79	0.32–1.96	0.618
Stains	0.37	0.18–0.76	**0.007**
Beta-blockers	0.77	0.52–1.15	0.199
ACEI/ARB	1.02	0.69–1.51	0.927
Hypoglycemic drugs	2.26	1.48–3.47	** < 0.001**
TyG index	1.96	1.41–2.73	** < 0.001**
TyG index (Per SD)	1.46	1.22–1.76	** < 0.001**

*MACE* major adverse cardiovascular events, *HR* Hazard ratio, *CI* Confidence interval, *BMI* body mass index, *LVEF* left ventricle ejection fraction, *MI* myocardial infarction, *GS* Gensini score, *FH-CAD* family history of coronary artery disease, *DM* diabetes mellitus, *FPG* fasting plasma glucose, TC total cholesterol, *TG* triglyceride, *LDL-C* low-density lipoprotein-cholesterol, HDL-C high-density lipoprotein-cholesterol, eGFR estimated glomerular filtration rate, UA uric acid, *ACEI* angiotensin-converting enzyme inhibitors, *ARB* angiotensin receptor blockers, *TyG* index triglyceride-glucose index, *SD* standard deviation

*p* values in bold are < 0.05

**Table 5:** Multivariate Cox regression analyses for MACE

TyG index	HR (95% CI)		
	**Model 1**	**Model 2**	Model** 3**
Per 1 Unit increase	1.97 (1.42–2.74)^**^	1.57 (1.07–2.29)^*^	1.94 (1.20–3.14)^*^
Per 1 SD increase	1.47 (1.22–1.76)^**^	1.29 (1.04–1.60)^*^	1.45 (1.11–1.91)^*^
Tertile 1	1 (Reference)	1 (Reference)	1 (Reference)
Tertile 2	1.94 (1.10–3.32)^*^	1.58 (0.91–2.75)	1.55 (0.87–2.78)
Tertile 3	2.70 (1.59–4.58)^**^	1.99 (1.12–3.52)^*^	2.17 (1.15–4.06)^*^
*p* for trend	** < 0.001**	**0.020**	**0.016**

Model 1: adjusted for age and gender

Model 2: adjusted for variables with *p*-value < 0.05 in univariate analysis, including BMI, multivessel disease, current smoking, DM, hypertension, LDL-C, stains and hypoglycemic drugs

Model 3: adjusted for age, gender, BMI, LVEF, admission for MI, multivessel disease, GS, current smoking, current drinking, FH-CAD, DM, hypertension, hyperlipidemia, TC, LDL-C, HDL-C, eGFR, UA, antiplatelet drugs, statins, beta-blockers, ACEI/ARB and hypoglycemic drugs

*MACE* major adverse cardiovascular events, *TyG index* triglyceride-glucose index, *HR* hazard ratio, *CI* confidence interval, *SD* standard deviation

*p* values in bold are < 0.05

^*^ *p* < 0.05

^**^ *p* < 0.001

We further studied the associations between the TyG index and non-fatal MI and coronary artery revascularization. The TyG index was found to be an independent risk factor for coronary artery revascularization rather than non-fatal MI (Table 6).

**Table 6:** Multivariate Cox regression analyses for coronary artery revascularization and non-fatal MI

TyG index	HR (95% CI)	
	Non-fatal MI	Coronary artery revascularization
Per 1 Unit increase	1.98 (0.80–4.90)	2.23 (1.20–4.14)^*^
Per 1 SD increase	1.47 (0.88–2.45)	1.57 (1.11–2.23)^*^
Tertile 1	1 (Reference)	1 (Reference)
Tertile 2	1.88 (0.59–5.92)	1.62 (0.77–3.42)
Tertile 3	1.85 (0.54–6.36)	2.76 (1.24–6.15)^*^
*p* for trend	0.364	**0.012**

Adjusted for age, gender, BMI, LVEF, admission for MI, multivessel disease, GS, current smoking, current drinking, FH-CAD, DM, hypertension, hyperlipidemia, TC, LDL-C, HDL-C, eGFR, UA, antiplatelet drugs, statins, beta-blockers, ACEI/ARB and hypoglycemic drugs

*MI* myocardial infarction, *TyG index* triglyceride-glucose index, *HR* hazard ratio, *CI* confidence interval, *SD*, standard deviation

*p* values in bold are < 0.05

^*^ *p* < 0.05

In addition, we analyzed the effect of DM on the occurrence of MACE. As shown in Table S2, DM was a risk factor for MACE [HR = 2.21 (1.45–3.37)] after adjusting for age and gender. These associations were, however, no longer significant after adjusting for other confounders (Additional file 1: Table S2).

### Subgroup analysis

**Fig. 3:**
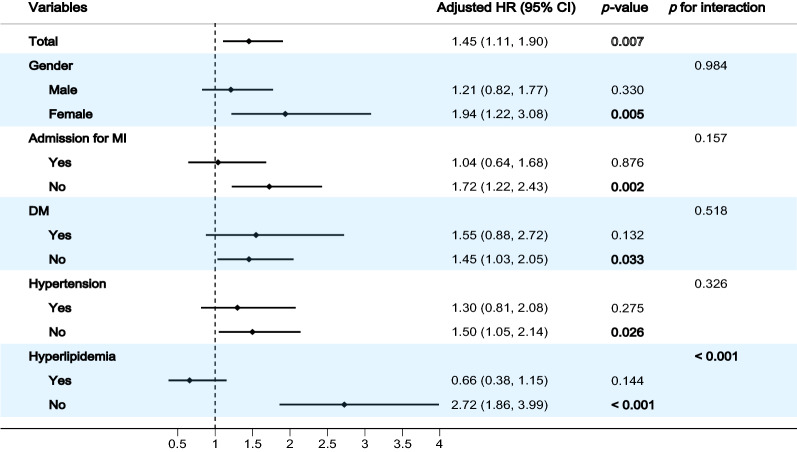
Subgroup and interaction analysis between the TyG index (Per SD) and MACE across various subgroups

The association between the TyG index and MACE was examined in the subgroup analysis. A significant interaction was found between hyperlipidemia and the TyG index for incident CVD (*p*-value for interaction < 0.001). Accordingly, a significant association between the TyG index and CVD was found only among patients without hyperlipidemia. Although no interaction was found between gender, admission for MI, DM, hypertension and the TyG index for incidence of MACE in multivariate analysis (All *p* -values for interaction $\ge$ 0.157), the statistical significance was observed only among females, patients admitted for non-MI, patients without DM and patients without hypertension (Fig. 3).

### Evaluation of the prognostic performance of the TyG index for MACE

**Fig. 4:**
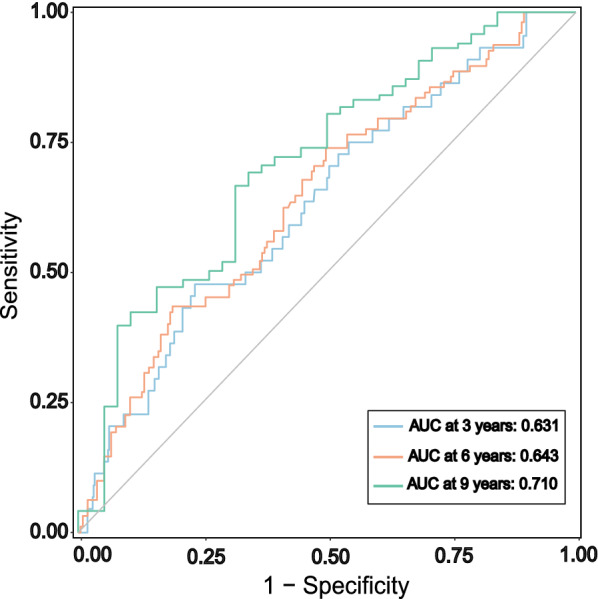
Time-dependent ROC curves of the TyG index for the prediction of MACE

To further evaluate the prognostic value and predictive performance of the TyG index, we performed time-dependent ROC analyses, and the area under the curves (AUC) reached 0.631 at 3 years, 0.643 at 6 years and 0.710 at 9 years (Fig. 4). The incremental predictive value of the TyG index for MACE was shown in Table 7.

**Table 7:** The incremental predictive value of the TyG index for MACE

	C-Statistic (95%CI)	*p*-value	Continuous NRI (95%CI)	*p*-value	IDI (95%CI)	*p*-value
Model 3 without TyG index	0.715(0.664–0.766)	Ref.		Ref.		Ref.
Model 3 with TyG index	0.719(0.666–0.772)	**0.007**	0.101 (-0.116–0.317)	0.362	0.011(0.002–0.021)	**0.017**

*TyG index* triglyceride-glucose index, *MACE* major adverse cardiovascular events, *NRI* net reclassification improvement, *IDI* integrated discrimination improvement, *Ref.* reference

*p* values in bold are < 0.05

According to C-statistic, risk prediction was improved by adding the TyG index to existing risk prediction model including age, gender, BMI, LVEF, admission for MI, multivessel disease, GS, current smoking, current drinking, FH-CAD, DM, hypertension, hyperlipidemia, TC, LDL-C, HDL-C, eGFR, UA, antiplatelet drugs, statins, beta-blockers, ACEI/ARB and hypoglycemic drugs (C-statistic increased from 0.715 to 0.719, *p* = 0.007). Otherwise, according to IDI, the TyG index significantly improved risk discrimination for MACE [IDI (95% CI): 0.011 (0.002–0.021), *p* = 0.017]. However, continuous NRI analysis did not show statistically significant improvement in classification [continuous NRI (95% CI): 0.101 (− 0.116–0.317), *p* = 0.362].

## Discussion

To the best of our knowledge, this was the first study to investigate the relationship between the TyG index and MACE in patients with PCAD. The main findings of our study were as follows: (1) The TyG index was associated with increased risk for MACE in PCAD patients, independent of traditional cardiovascular risk factors. (2) The significant association between the TyG index and MACE was mainly observed among females, patients admitted for non-MI, and patients without DM, hypertension or hyperlipidemia. (3) Adding the TyG index to the existing risk prediction model could improve outcome prediction in patients with PCAD. Taken together, our findings revealed the prognostic value of the TyG index for MACE in patients with PCAD.

Although coronary artery disease is the main underlying disease primarily in the elderly, CAD in young individuals has become more common in recent years and is often associated with poor outcomes [3, 4]. However, there is insufficient research about the risk factors of PCAD, and previous studies have focused mainly on traditional cardiovascular risk factors [5, 6]. Insulin resistance (IR) is a general term meaning that adipose tissue, skeletal muscle, liver and pancreas display a low response to insulin action. Theoretically, IR can aggravate atherosclerosis by systemic inflammation, endothelial dysfunction and oxidative stress [28, 29], and IR has been proven a risk factor for cardiovascular disease [10, 11]. The Hyperinsulinemic-euglycemic clamp technique is the gold standard to assess IR and HOMA-IR is the most widely used method. However, the hyperinsulinemic-euglycemic clamp technique is costly and time‐consuming [13], whereas HOMA-IR is likely to cause significant bias because of insulin measurements [30, 31]. In this regard, the TyG index, as a simple surrogate for IR, has proven to be of prognostic value for CAD [21, 22]. The prognostic value of the TyG index for patients with PCAD remains, however, poorly known.

In the present study, our results revealed the correlations between the TyG index and other risk factors. Previous studies also revealed the relationships between the TyG index and obesity, dyslipidemia and renal insufficiency [32–34]. More importantly, we reported for the first time the positive correlation between the TyG index and the severity of PCAD assessed by GS. Given the low acceptance of coronary angiography in young patients, the TyG index could be a complementary evaluation method of coronary lesion severity among young people.

The incidence of adverse events was lower in this cohort of young people than that in patients of all ages [35]. The relatively low incidence of all-cause death and non-fatal stroke was consistent with the previous study [6]. Zhu Y et al. retrospectively recruited patients who were admitted for acute coronary syndrome (ACS) and underwent PCI and found that the TyG index was associated with in-stent restenosis [21]. Jiao Y et al. found that the TyG index was an independent predictor of all-cause mortality and MACE in a cohort study including 662 elderly patients with ACS [22]. The prognostic value of TyG in young patients was first discovered in this study.

DM patients usually suffer from one or more components of metabolic syndrome. Intensified multifactorial intervention can reduce the risk of death from cardiovascular causes and of cardiovascular events [36–39]. In this study, most patients were on standardized antidiabetic, antihypertensive and lipid-lowering therapy. Intensified multifactorial intervention might be one of the reasons why DM was not an independent risk factor of MACE.

The sex differences in the risk of cardiovascular disease associated with IR have been reported before. A meta-analysis included 87 studies found that females with metabolic syndrome had a higher risk of CVD than males [11]. The Framingham study also showed females with impaired fasting glucose had increased CVD risk to a similar degree as established diabetes, but not in males [40]. This excess risk in females could be due to sex differences in the body anthropometry, preferred location of fat storage^41^, heavier risk factor burden and a greater effect of a higher TG and blood pressure [42]. These epidemiological findings and mechanism researches could partly explain the sex differences in the prognostic value of the TyG index. For part of patients with MI, the level of FPG was elevated, which was called “stress hyperglycemia” [43]. In such patients, the TyG index, combining FPG and TG, was no longer able to accurately reflect the degree of insulin resistance, and as a result, diminished the prognostic value for MACE. In patients without DM, hypertension and hyperlipidemia, a significant association between the TyG index and MACE was observed in this study, which suggested that the TyG index might be independent of DM, hypertension and hyperlipidemia to influence cardiovascular outcomes. A previous study also reported the independent prognostic value of the TyG index in NSTE-ACS patients without glucose metabolism disorder [44]. However, the association between the TyG index and MACE was not statistically significant in patients with hypertension, DM and hyperlipidemia. Different antihypertensive drugs may have different effects on insulin action and lipid levels [45], thereby affecting the TyG index. In patients with DM and hyperlipidemia, the TyG index cannot be accurately calculated because of the use of hypoglycemic agents, lipid-lowering agents and many other drugs which can affect the lipid panel or level of glucose. Moreover, in patients with T2DM, the classic CVD risk factors are major predictors of CVD events, and the risk is further increased by hyperglycemia, but to a lesser extent as by insulin resistance alone [28]. These causes may partly explain the differences in the predictive power of the TyG index we observed here.

Previous studies showed the addition of the TyG index to the baseline risk model could improve the MACE prediction in patients after PCI and elderly ACS patients [21, 22]. In our study, we found that the addition of the TyG index has a significant incremental prognostic value for predicting MACE in patients with PCAD.

Several limitations of this study should be considered. First, this was a single-center retrospective study, so potential bias could have been introduced. Second, the sample size was relatively small, and the incidence of all-cause death and non-fatal stroke was relatively low, which might limit a sound statistical analysis and made it difficult to elaborate the associations between the TyG index and the individual components of MACE. Third, the insulin levels were not measured in most patients in this study and the HOMA-IR values cannot be calculated. Fourth, laboratory parameters were only detected once at admission with a potential bias due to measurement error. Finally, we did not record nutritional habits and physical activities, which might affect the TyG indexes. Further multicenter, large-size, prospective studies may strengthen our conclusion.

## Conclusion

In conclusion, the present data demonstrate that the TyG index is significantly associated with the severity of PCAD, and is a valuable predictor of MACE in PCAD patients. Therefore, we propose that the TyG index is a simple and reliable index for the risk stratification and early intervention of PCAD.

## Supplementary Information

**Additional file 1: Table S1.** Sensitivity analysis for the association between the TyG index and MACE. **Table S2**. Multivariable Cox regression analyses for the association between DM and MACE.

## Data Availability

The datasets used and/or analyzed during the current study are available from the corresponding author on reasonable request.

## Abbreviations

PCAD: Premature coronary artery disease
TyG index: Triglyceride-glucose index
IR: Insulin resistance
MACE: Major adverse cardiovascular event
GS: Gensini score
AUC: Area under the curve
ROC: Receiver operating characteristic
NRI: Net reclassification improvement
IDI: Integrated discrimination improvement
CAD: Coronary artery disease
TLGS: Tehran Lipid and Glucose Study
ACS: Acute coronary syndrome
LM: Left main coronary artery
LAD: Left anterior descending artery
LCX: Left circumflex coronary artery
RCA: Right coronary artery
BMI: Body mass index
LVEF: Left ventricular ejection fraction
FH-CAD: Family history of CAD
DM: Diabetes mellitus
FPG: Fasting plasma glucose
TC: Total cholesterol
TG: Triglyceride
LDL-C: Low‐density lipoprotein cholesterol
HDL-C: High-density lipoprotein cholesterol
SCr: Serum creatinine
UA: Uric acid
ACEI: Angiotensin-converting enzyme inhibitors
ARB: Angiotensin receptor blockers
MI: Myocardial infarction
RBG: Random blood glucose
OGTT: Oral glucose tolerance test
eGFR: Estimated glomerular filtration rate
VIF: Variance inflation factor
SD: Standard deviation
HR: Hazard ratio
CI: Confidence interval
