# Supplementary material for: Triglyceride-glucose index in the prediction of adverse cardiovascular events in patients with premature coronary artery disease: a retrospective cohort study
Source: Cardiovasc Diabetol. 2022 Jul 29;21:142. doi: 10.1186/s12933-022-01576-8 (PMC9338459; doi:10.1186/s12933-022-01576-8)
Supplement: Supplementary file 1 — Additional file 1: Table S1. Sensitivity analysis for the association between the TyG index and MACE. Table S2. Multivariable Cox regression analyses for the association between DM and MACE. [file 12933_2022_1576_MOESM1_ESM.docx]

**Table S1 Sensitivity analysis for the association between the TyG index and MACE**

| **TyG index** | **HR (95% CI)** | |
| --- | --- | --- |
|  | **Analysis 1** | **Analysis 2** |
| Per 1 Unit increase | 3.20 (1.56-6.58) **^*^** | 1.99 (1.22-3.23) **^*^** |
| Per 1 SD increase | 1.93 (1.28-2.89) **^*^** | 1.47 (1.12-1.94) **^*^** |
| Tertile 1 | 1 (Reference) | 1 (Reference) |
| Tertile 2 | 2.04 (0.99-4.18) | 1.61 (0.89-2.91) |
| Tertile 3 | 2.60 (1.14-5.96) **^*^** | 2.24 (1.18-4.23) **^*^** |
| *p* for trend | **0.016** | **0.013** |

Analysis 1: Excluding patients with a history of lipid-lowering or hypoglycemic usage.

Analysis 2: Excluding non-cardiovascular death.

Model adjusted for age, gender, BMI, LVEF, admission for MI, multivessel disease, GS, current smoking, current drinking, FH-CAD, DM, hypertension, hyperlipidemia, TC, LDL-C, HDL-C, eGFR, UA, antiplatelet drugs, statins, beta-blockers, ACEI/ARB.

MACE, major adverse cardiovascular events; TyG index, triglyceride-glucose index; HR, Hazard ratio; CI, Confidence interval; SD, standard deviation.

* *p* < 0.05

*p* values in bold are < 0.05.

**Table S2 Multivariable Cox regression analyses for the association between DM and MACE**

|  | **HR (95% CI)** | ***p-*value** |
| --- | --- | --- |
| **Model 1** | 2.21 (1.45-3.37) | **< 0.001** |
| **Model 2** | 1.28 (0.39-4.19) | 0.680 |
| **Model 3** | 0.97 (0.29-3.27) | 0.964 |

Model 1: adjusted for age and gender.

Model 2: adjusted for variables with *p*-value <0.05 in univariate analysis, including BMI, multivessel disease, current smoking, hypertension, LDL-C, stains and hypoglycemic drugs.

Model 3: adjusted for age, gender, BMI, LVEF, admission for MI, multivessel disease, GS, current smoking, current drinking, FH-CAD, hypertension, hyperlipidemia, TC, LDL-C, HDL-C, eGFR, UA, antiplatelet drugs, statins, beta-blockers, ACEI/ARB and hypoglycemic drugs.

DM, diabetes mellitus; MACE, major adverse cardiovascular events; HR, hazard ratio; CI, confidence interval.

*p* values in bold are < 0.05.
